# Intraosseous Hemangioma in the Humerus Diaphysis in an Eight-Year-Old Girl

**DOI:** 10.7759/cureus.17375

**Published:** 2021-08-23

**Authors:** Recep Ozturk, emin kürşat bulut

**Affiliations:** 1 Orthopedics and Traumatology, Dr Abdurrahman Yurtarslan Ankara Oncology Education and Research Hospital, Ankara, TUR

**Keywords:** intraosseous hemangioma, bone, hemangioma, child, bone hemangioma

## Abstract

In this study, we present an eight-year-old patient with intraosseous hemangioma in the right humerus diaphysis. The humerus diaphysis is an unusual localization for hemangioma. To our knowledge, this is the first case of intraosseous hemangioma in the humerus diaphysis in a pediatric patient.

Treatment of intraosseous hemangiomas is controversial; options range from untreated follow-up to en-bloc resection. Intralesional curettage and grafting with cortico-cancellous allograft were performed in this case. Around 22 months postoperatively, she showed full shoulder and elbow function and there was no evidence of local recurrence or metastasis.

## Introduction

Intraosseous hemangiomas (IHs) are rare benign vascular tumors and account for less than 1% of all bone tumors. In Turkey, there are publications reporting that it is seen less than one percent [1]. It is usually found in the vertebrae, but also in the calvarium. It is rarely seen in long bones, tubular bones, or ribs [2]. Lesions in long bones are usually found in metaphysis or diaphysis [3].

Most bone hemangiomata are seen on radiographic findings with the formation of reactive spicules produced by the lesion [4]. Clinical presentation may vary from incidental asymptomatic lesions to pain or pathological fractures, depending on lesion size and localization [5]. The diaphysis of the humerus is a very rare localization for the appearance of this tumor, so it makes it difficult to diagnose. Oncologists specializing in orthopedics do not have much experience in the treatment of a solitary hemangioma in long bone diaphysis [6].

Because of the variable appearance of the lesion, histological examination is vital to obtain a definitive diagnosis. Other benign and malignant tumors (fibrous dysplasia, eosinophilic granuloma, aneurysmal bone cyst, Ewing's sarcoma, chondrosarcoma, metastasis) should be considered in the differential diagnosis [7].

There are only a few case presentations reporting hemangiomas localized in the humerus. Moreover, humeral diaphysis is a very rare localization [3,6,8,9]. We found hemangioma located in isolated humerus diaphysis only in three adult patients [3,6,9]. To our knowledge, this is the first reported case of isolated humeral diaphysis in a pediatric patient.

## Case presentation

An eight-year-old girl was admitted to our orthopedics department with a complaint of pain in her right arm midline. She said her complaints had been going on for six months and getting worse. In addition, the patient could not recall any trauma and did not have any systemic or metabolic disease. The study was conducted in accordance with the principles of the Declaration of Helsinki.

**Figure 1:**
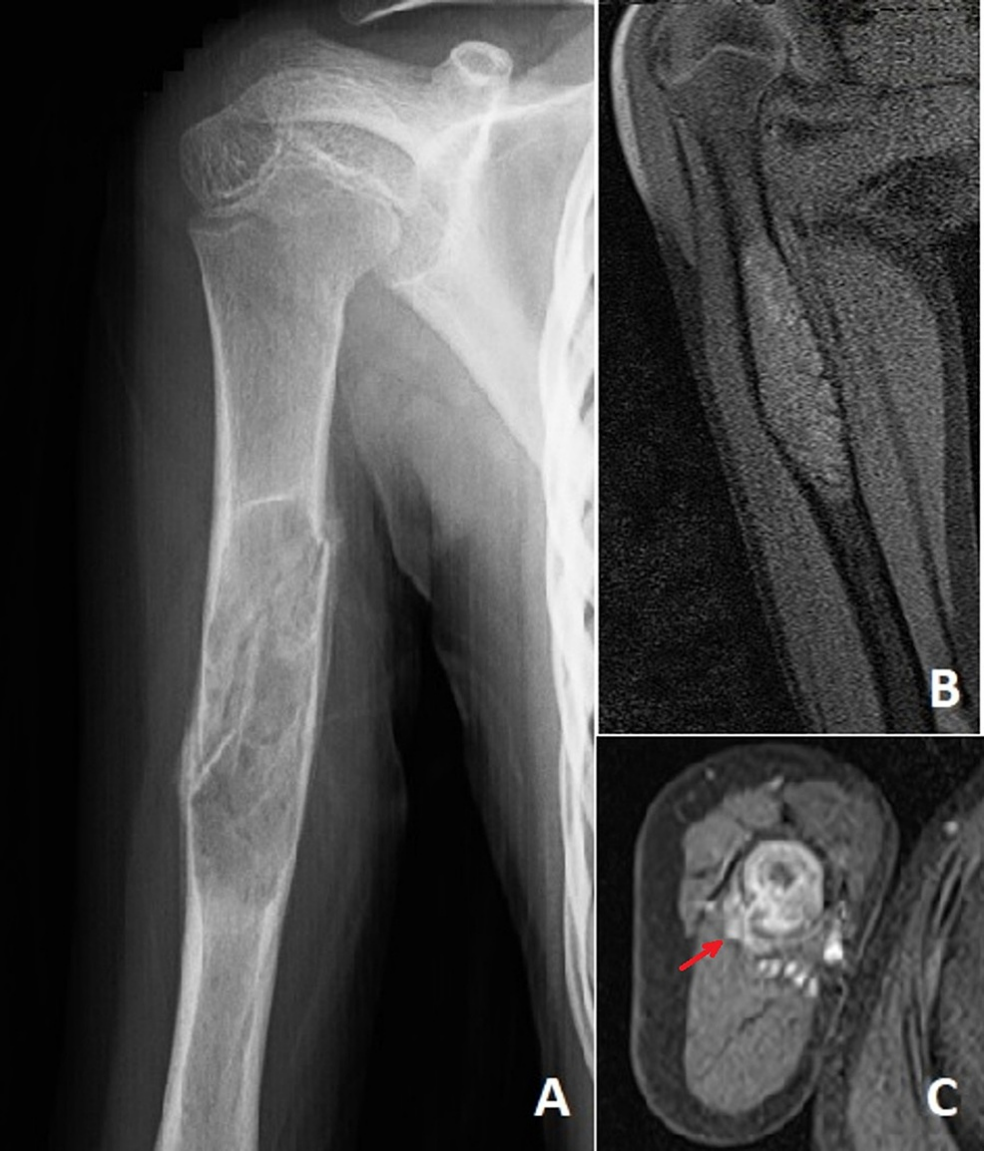
A: Anteroposterior radiography of the right humerus diaphysis with foamy patchy lytic areas of the expansile lesion that caused a fracture. B: T1-weighted coronal MRI showed a medullary lesion without soft tissue component thinning the hypointense expansile cortex. C: T2-weighted axial image showed a heterogeneous hyperintense intramedullary lesion with mild contrast enhancement in the posterolateral parts of the bone (arrow).

On the clinical examination, pain was detected in the midline of the right arm, but shoulder and elbow joint movements were regular and painless. On the plain radiograph, there was a smooth lesion with a narrow transitional zone and foamy patchy lytic areas in the humeral diaphysis leading to expansion of the central bone. Within the lesion, a linear lucent line was observed, which was consistent with the fracture leading to a smooth periosteal reaction, extending obliquely to the medial and lateral cortex (Figures 1A-1B).

The MRI revealed a 72 × 21x21 mm mass in the right humeral diaphysis with an intramedullary expansile nature. In T1 and T2 MRI-weighted series, the heterogeneous density of the mass and contrast enhancement after intravenous gadolinium injection are noteworthy. In addition, thinning of the cortex, patchy irregularities, and defective view suggested pathologic fractures. In addition to the described lesion, contrast enhancement was observed in the soft tissues after intravenous injection of contrast material in the posterolateral sections (Figures 1B-1C).

**Figure 2:**
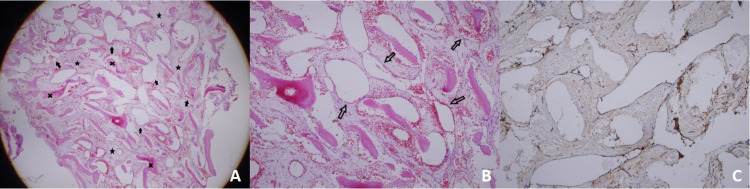
A histological examination of the existed tissue confirmed the typical features of the hemangioma Figure 2A (4x10 H&E image) -  X: Lamellar bone trabeculae. Arrows: enlarged vascular structures including erythrocytes. Star: Fibrous bands. Figure 2B (20x10 H&E image) -  Arrows: Dilated thin-walled vascular structures including erythrocyte aggregates. Figure 2C: Factor 8 Immunohistochemical examination depicting vascular structures appearing as brown lines, which represent cytoplasmic staining of vascular endothelium.

**Figure 3:**
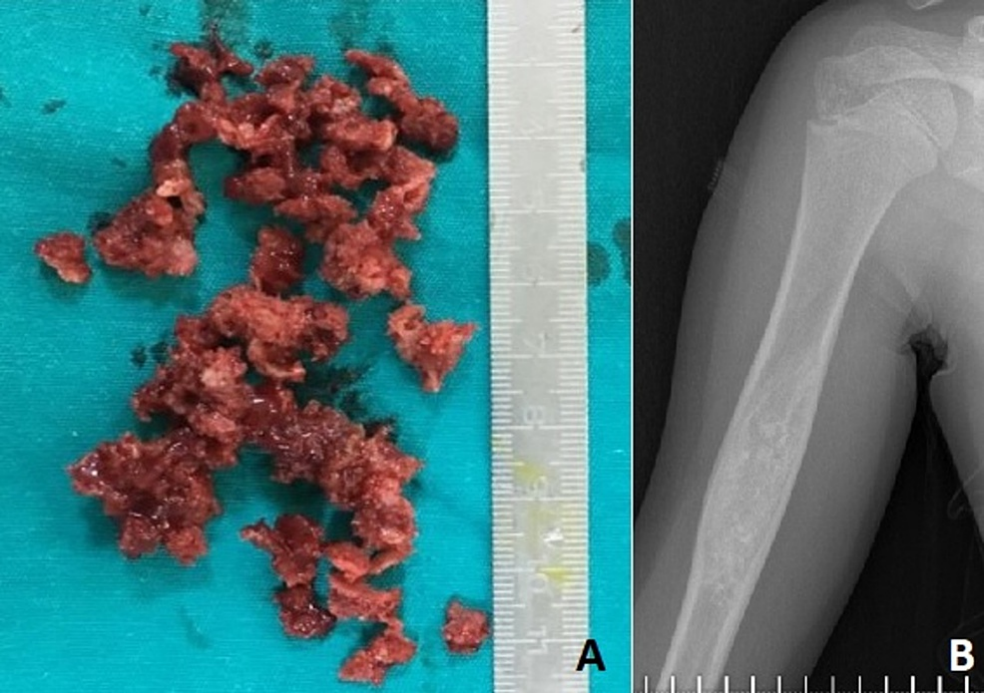
Macroscopic view of the excised lesion (A); Postoperative third-month control X-ray after curettage and bone grafting (B).

Incisional biopsy was performed and intraosseous hemangioma was shown. Histological examination of the excised tissue confirmed the typical features of the hemangioma (Figures 2A-2C). The differential diagnosis from aneurysmal bone cyst was made by the presence of widely dilated vessels, thick vessel walls, and absence of cellular blood-filled spaces. Based on the diagnosis, we performed marginal excision of the tumor, then grafted it with approximately 30 cc. allogeneic cortico-cancellous graft (Figures 3A-3B).

No intraoperative or postoperative complications were observed. At 24 months of follow-up, the patient did not feel any pain and there was a full range of motion in the shoulder and elbow.

## Discussion

IHs constitute <1.0% of all bone tumors; moreover, 1. 75% of tumors are localized in the vertebral body and skull. In 15-20% of all cases, tumors are found in the scapula, ribs, clavicle, and pelvic bones [7]. Its settlement in long bones is very rare and tends to settle in the lower limbs in long bones [3,10]. The humeral diaphysis is extraordinary. To our knowledge, it was found only in three adult patients [3,6,9].

IHs are typically seen in adult patients in the third and fourth decades of life, thus very rarely seen in skeletally immature patients younger than 10 years. In their literature review, Andreciccio et al. found that there were 14 cases, including his own, in 2007 [7].

Although IH is the most common solitary lesion, approximately 18-25% of IH cases are multifocal [11]. IH with the appendicular skeleton is usually symptomatic, as opposed to the axial skeleton, which is usually asymptomatic [12]. In our case, the patient presented with pain symptoms.

Plain radiographs show a classical coarse trabecular bone pattern or soap bubble appearance. Most lesions show cortical destruction [13]. On plain X-ray and computed tomography, the lesion is usually well-circumscribed [11]. On MRI, the lesion may have different appearances. Generally, T1-weighted sequences may show hypo or hyper-intensities depending on the amount of fat contained, and T2-weighted sequences show hyperintense images due to vascularity [14].

Radiographic diagnosis of an atypical intraosseous hemangioma is more difficult due to various possible diagnoses such as fibrous dysplasia, solitary or aneurysmal bone cysts, eosinophilic granuloma, multiple myeloma, chondrosarcoma, Ewing’s sarcoma, metastasis, other benign and malignant lesions [15].

The biopsy will be the gold standard to obtain the correct diagnosis [15]. Needle biopsy or Tru-cut biopsy is essential for diagnosis, but it may be more appropriate to avoid biopsy due to the risk of bleeding in large hemangiomas [6]. In this case study, we preferred to perform an open incisional biopsy to avoid possible misdiagnosis.

Histopathological examination of intraosseous hemangioma reveals thin-walled blood vessels. If these structures are damaged during biopsy or curettage, non-diagnostic empty spaces may be encountered and only bone trabeculae are detected. Therefore, an intraosseous hemangioma may pose a diagnostic challenge for the pathologist 3. In our study, there were sections containing bone trabeculae, and the diagnosis was made by the presence of dilated thin-walled vascular structures containing erythrocytes. The differential diagnosis of an aneurysmal bone cyst was made with the absence of cellular blood-filled spaces and fibroblastic cells.

The treatment of intraosseous hemangioma is a controversial subject ranging from biopsy to segmental resection [6,11]. Small asymptomatic long bone IHs usually do not usually require surgical treatment. Large, symptomatic lesions or pathological fractures are usually treated with curettage and bone grafting. However, there are also reports indicating that radical en bloc resection should be performed to avoid recurrence and massive bleeding. We preferred curettage and bone grafting in the pediatric patient and received good results.

## Conclusions

This is the first report of an intraosseous hemangioma of the humeral diaphysis in a pediatric patient. After obtaining the histopathological diagnosis by biopsy, it was successfully treated with curettage and bone grafting.
